# Mutational spectrum of *EDA*, *EDAR*, *EDARADD*, and *WNT10A* genes in the largest cohort of Russian patients with hypohidrotic ectodermal dysplasia

**DOI:** 10.1186/s13023-026-04211-x

**Published:** 2026-02-05

**Authors:** Valeriia A. Kovalskaia, Tatiana B. Cherevatova, Elena V. Zinina, Olga A. Shagina, Ekaterina O. Vorontsova, Galina N. Matyushchenko, Nina A. Demina, Marina P. Petukhova, Tatiana V. Markova, Daria M. Guseva, Varvara A. Galkina, Inga V. Anisimova, Anna A. Stepanova, Alena L. Chuhrova, Margarita V. Sharova, Fatima M. Bostanova, Anahit E. Voskanyan, Aleksander V. Polyakov, Oxana P. Ryzhkova

**Affiliations:** Research Centre for Medical Genetics, Moscow, 115522 Russia

**Keywords:** *EDA*, *EDAR*, *EDARADD*, *WNT10A*, Ectodermal dysplasia, Hypohidrotic ectodermal dysplasia, HED, Mutational spectrum

## Abstract

**Background:**

Hypohidrotic ectodermal dysplasia (HED) encompasses a group of rare genetic disorders affecting two or more ectodermal derivatives (hair, teeth, nails, certain glands). The condition can be inherited in an X-linked, autosomal dominant, or autosomal recessive manner, with the majority of cases caused by mutations in the *EDA*,* EDAR*,* EDARADD*, and *WNT10A* genes. This study aimed to evaluate the distribution of pathogenic and likely pathogenic variants in 261 unrelated families affected by HED in the Russian Federation (comprising 455 patients in total) between 2007 and 2024. To achieve this objective, we employed Sanger sequencing, targeted gene panel sequencing (NGS), multiplex ligation-dependent probe amplification (MLPA), and segregation analysis to clarify the pathogenicity of variants of uncertain significance.

**Results:**

A total of 261 unrelated probands, comprising 196 males (75.1%) and 65 females (24.9%), were included. Pathogenic or likely pathogenic variants were identified in 183 probands (70.1%). The distribution of mutated genes was as follows: *EDA* (*n* = 155, 84.7%), *WNT10A* (*n* = 16, 8.8%), and *EDAR* (*n* = 12, 6.5%). No apparent pathogenic mutations were detected in *EDARADD*. Additionally, we report 46 novel causative variants for HED, along with recurrent mutations in the *EDA*,* WNT10A*, and *EDAR* genes. We also identified that 28.8% of all causative variants in *EDA* are *de novo*.

**Conclusion:**

This is the only molecular study conducted in the Russian population affected by HED and represents the largest HED cohort published to date globally. Our findings significantly expand the mutational spectrum of HED-causing genes and will aid in choosing an initial diagnostic approach for HED patients. Further studies using whole-genome sequencing (WGS) will help to identify other contributory genes in the remaining uncharacterized Russian patients with HED.

**Supplementary Information:**

The online version contains supplementary material available at 10.1186/s13023-026-04211-x.

## Background

The term “ectodermal dysplasia (ED)” refers to a diverse group of genetic disorders marked by abnormalities in the development and/or homeostasis of two or more ectodermal structures [1]. While the ectoderm is responsible for the formation of various organs and tissues, including the central and peripheral nervous systems, pituitary gland, olfactory neuroepithelium, melanocytes, as well as tooth, epidermis, sweat glands, hair, and nails, ED predominantly affects only the latter set of ectodermal derivatives.

The first recorded case of ED dates back to 1838, when Wedderburn described a family of 10 Indian men with partial tooth agenesis, alopecia, and excessively dry skin in a letter to Charles Darwin [2]. In 1929, Weech first introduced the concept of “hereditary ectodermal dysplasia” and established the term “anhidrotic,” which was later revised to “hypohidrotic,” to describe individuals with this condition characterized by a diminished ability to sweat [3].

Thus, patients with hypohidrotic ectodermal dysplasia (HED) present hallmark features, including abnormalities of hair (scalp hypotrichosis/alopecia, sparse-absent eyebrows/eyelashes), teeth (anodontia/ oligodontia/ microdontia/ conical teeth/ misshapen teeth), nails (hypoplastic, misshapen nails), skin (dry, hyperpigmented) and sweat glands (anhidrosis or hypohidrosis), as well as additional signs, such as frontal bossing, hypoplastic mammary glands, eczema, prominent lips, etc.

The overall global prevalence of ED has not been precisely established due to the limited number of studies and the absence of a consistent classification system across different countries. However, this number is estimated to be approximately 7 per 10,000 live births [4]. The estimated minimum prevalence of X-linked ectodermal dysplasia (XLHED) ranges from 2.8 to 2.99 per 100,000 [5, 6]. Consequently, although EDs are not among the most common hereditary disorders, they are nonetheless widespread and contribute significantly to dental, dermatological, and genetic pathologies.

**Fig. 1:**
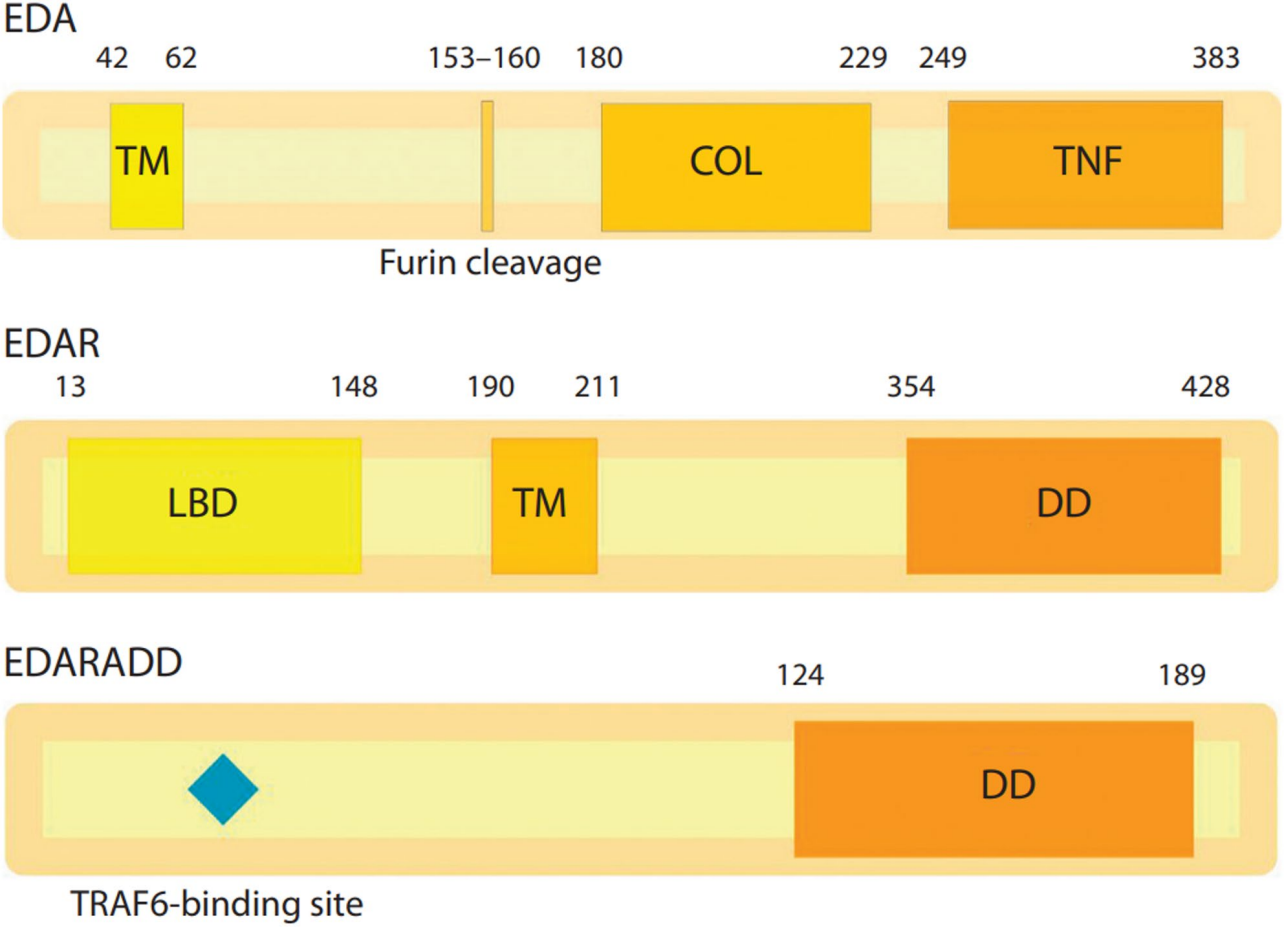
Domain structure of the EDA, EDAR and EDARADD proteins [7, 8]. TM – transmembrane domain; Furin cleavage – furin cleavage site; COL – collagen domain; TNF – tumor necrosis factor domain; LBD – ligand-binding domain; DD – death domain

The most common HED type is XLHED, which is caused by pathogenic and likely pathogenic variants in the *EDA* gene (OMIM 305100). The ectodysplasin A (*EDA*) gene, located on the X chromosome at the Xq13.1 locus, encodes 8 protein-coding isoforms. Among these proteins, only isoform 1 (EDA-A1), a 391-amino acid protein, serves as the ligand for the EDAR receptor (Fig. 1). The EDAR protein is encoded by the gene of the same name located at chr2q12.3. It interacts with the EDARADD death domain, initiating downstream NF-κB signaling. This signaling cascade ultimately regulates the expression of genes essential for epidermal, hair, tooth, and nail development [9, 10]. Both the *EDAR* and *EDARADD* genes are implicated in autosomal recessive (OMIM 224900, OMIM 614941) and autosomal dominant (OMIM 129490, OMIM 614940) forms of ED. Interestingly, pathogenic mutations in *EDA*,* EDAR*, and *EDARADD* can result in phenotypically similar manifestations, posing significant challenges for clinical differentiation.

Mutations in the *WNT10A* gene are recognized as the primary cause of non-syndromic selective tooth agenesis (OMIM 150400) [11]. However, its biallelic mutations have also been associated with HED, odontoonychodermal dysplasia (OODD) (OMIM 257980) and Schöpf-Schulz-Passarge syndrome (OMIM 224750). The *WNT10A* gene encodes a protein involved in the canonical Wnt/β-catenin signaling pathway, which plays a crucial role in various aspects of tooth morphogenesis. Specifically, this pathway activates mesenchymal odontogenic potential during early tooth development and is essential for the induction and maintenance of primary and secondary enamel knots. Additionally, the role of Wnt signaling in the morphogenesis of hair follicles and skin structures remains an area of active investigation [12].

The objective of this study was to evaluate, for the first time, the mutational spectrum of the *EDA*,* EDAR*,* EDARADD*, and* WNT10A* genes in 261 Russian patients exhibiting clinical features of HED, which is crucial for the development of diagnostic approaches. This study includes the largest known cohort of individuals with HED published to date worldwide.

## Materials and methods

### Patients

This study enrolled 455 patients from 261 unrelated families, with at least one family member clinically diagnosed with HED between 2007 and 2024. All 455 patients underwent genetic testing. Most patients were examined directly by clinical geneticists at the Research Centre of Medical Genetics (RCMG, Moscow, Russia). For the others, data was collected via a questionnaire that gathered clinical information and family history.

Patients were included in the cohort if they had either two or more abnormalities in ectodermal derivatives or had been referred for laboratory testing with a diagnosis of HED. We excluded patients who had syndromic disorders with HED-like features (e.g., AEC syndrome (OMIM 106260) or Goltz–Gorlin syndrome (OMIM 305600)) or who had just non-syndromic tooth agenesis (NSTA).

Written informed consent was obtained from all participants, or from parents in the case of minors, prior to blood sample collection for genetic analysis. The study protocol was approved by the Ethical Committee of the Research Centre for Medical Genetics (Moscow, Russia) and followed the ethical standards of the Helsinki Declaration. Genomic DNA was extracted from fresh peripheral blood samples via the Wizard^®^ Genomic DNA Purification Kit (Promega Corporation, USA).

### Sanger sequencing and NGS

The first step of the study involved Sanger sequencing (Applied Biosystems, USA) of the *EDA* gene. To cover all 8 coding exons and exon‒intron boundaries of *EDA* (NM_001399), specific primers were designed and are available upon request. The identified *EDA* variants were subsequently subjected to segregation analysis within the families whenever possible.

In cases where pathogenic variants in *EDA* were not detected, custom next-generation sequencing (NGS) gene panel testing was performed. This panel included the entire coding sequences of *EDA*,* EDAR*,* EDARADD*,* GJB6*,* WNT10A*,* WNT10B*,* CST6*,* GREM2*,* HOXC13*,* KDF1*,* KREMEN1*,* KRT74*,* KRT85*,* LRP6*,* MSX1*,* PAX9*,* TSPEAR*, and *TP63*. DNA sequencing was conducted via an Illumina MiSeq sequencer with a MiSeq Reagent Kit v2 (500 cycles). Standard automated algorithms provided by Thermo Fisher Scientific (Torrent Suite™) and NGS-Data software (Russia, Moscow, 2021.1) were used for processing the sequencing data. Variants were described according to HGVS nomenclature, with MANE Select reference sequences. To estimate the population frequencies of identified variants, the gnomAD (V3.1.2) and RuExAc databases were consulted. RuExAc is based on 2910 exome sequences from Russian patients with various hereditary diseases collected by the Research Centre for Medical Genetics. Filtered variants were further analyzed via *in silico* pathogenicity prediction tools, including REVEL, PolyPhen-2, SIFT, PROVEAN, M-CAP, Mutation Assessor, Mutation Taster, SpliceAI, and Human Splicing Finder, to assess their potential impact. The clinical relevance of the identified variants was evaluated via the databases HGMD^®^ (2025.1) and LOVD, as well as relevant literature data.

### MLPA-based assessment of copy number variations

If a causative mutation was not identified, multiplex ligation-dependent probe amplification (MLPA) analysis was performed using the SALSA^®^ MLPA^®^ Probemix P183 EDA-EDAR-EDARADD commercial kit (MRC-Holland, Amsterdam, Netherlands) according to the manufacturer’s instructions. This assay was used to detect gross deletions and duplications. Fragment sizes were determined via capillary electrophoresis. Exon copy numbers were assessed based on peak height values and analyzed using Coffalyser^®^ software (MRC-Holland, Amsterdam, Netherlands).

### X-chromosome inactivation (XCI) patterns

X chromosome inactivation (XCI) patterns were analyzed using an assay targeting the polymorphic CAG repeat in exon 1 of the androgen receptor (AR) gene. Peripheral blood DNA was digested with the methylation-sensitive restriction enzyme HpaII, which cleaves restriction sites only on active X chromosomes, leaving inactive (methylated) X chromosomes intact. Following DNA digestion, PCR amplification was performed via a fluorophore-labeled forward primer, and fragment analysis was conducted on a 3130xl Genetic Analyzer (HITACHI, Applied Biosystems, USA/Japan). X-chromosome inactivation patterns were classified as random if the ratio was 75:25 or less, moderately skewed if it was between 75:25 and 80:20, and highly skewed if it was greater than 80:20.

### Analysis of mutations` origin (inherited or *de novo*)

To determine whether the identified variants were inherited or *de novo*, genotyping of DNA samples from the probands and their parents was performed. This was achieved by amplifying 16 human loci via the AmpFlSTR^®^ Identifiler^®^ Direct PCR Amplification Kit (Applied Biosystems, LLC, USA), following the manufacturer’s protocol. The amplification products were then separated by capillary electrophoresis on the aforementioned analyzer.

## Results

**Fig. 2:**
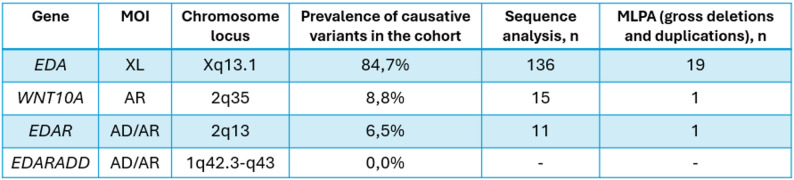
Distribution of pathogenic and likely pathogenic variants in HED-associated genes in the cohort

From 2007 to 2024, a total of 455 patients from 261 unrelated families were referred for genetic testing, 24.9% (*n* = 65) of the probands were female, whereas rest probands 75.1% were males (*n* = 196). We also analyzed the ethnic background of the people in our cohort. The majority of the patients identified themselves as Russians (70.91%), whereas other ethnicities were significantly less represented, including Tatars, Dagestanis, Bashkirs, Armenians, and others.  In a cohort of 261 unrelated patients, 214 genetic variants were identified, 183 (70.11%) of which were classified as causative, i.e., pathogenic or likely pathogenic, according to ACMG criteria [13] (Fig. 2). Among them, 46 variants were considered novel and previously unreported. The remaining 30 mutations were defined as variants of uncertain clinical significance (Table 1), and further investigation is needed to determine their pathogenicity.

**Table 1:** Likely causative VUS in HED/NSTA-associated genes

Gene	VUS variant	MOI	State	Times detected	ACMG criteria
*EDA*	c.194G > C (p.Arg65Pro)	XL	Hemi	1	PM2, PM5, PP3
*EDA*	c.395_396AG > CC (p.Gln132Pro, splicing?)	XL	Hetero	1	PM2, PP3
*EDA*	c.397–3C > G (splicing?)	XL	Hemi	1	PM2, PP3
*EDA*	c.680G > C (p.Gly227Ala)	XL	Hemi	1	PM2, PP3
*EDA*	c.706G > A (p.Gly236Ser, splicing?)	XL	Hemi	1	PM2, PP3
*EDA*	c.707-3del (splicing?)	XL	Hemi	1	PM2, PP3
*EDA*	c.776_784 (p.Ala259_Gln261del)	XL	Hemi	1	PM2, PM4, PP3
*EDA*	c.782 A > C (p.Gln261Pro)	XL	Hemi	1	PM2, PP3
*EDA*	c.806G > A (p.Gly269Glu)	XL	Hemi	1	PM2, PP3
*EDA*	c.872G > T (p.Gly291Val)	XL	Hemi	1	PM2, PP3, PM5
*EDA*	c.908T > A (p.Ile303Asn)	XL	Hemi	1	PM2, PP3
*EDA*	c.924 + 6T > C (splicing?)	XL	Hetero	1	PM2, PP3
*EDA*	c.927_935del (p.Tyr310_Ile312del)	XL	Hemi	1	PM2, PP3, PM4
*EDA*	c.929 A > G (p.Tyr310Cys)	XL	Hemi	1	PM2, PP3
*EDA*	c.952_953GC > AA (p.Ala318Thr)	XL	Hemi	1	PM2, PP3
*EDA*	c.952G > C (p.Ala318Pro)	XL	Hemi	1	PM2, PP3
*EDA*	c.971_973del (p.Val324del)	XL	Hemi	1	PM2, PP3
*EDA*	c.1033 A > G (p.Thr345Ala)	XL	Hemi	1	PM2, PP3
*EDA*	c.1060 C > T (p.Leu354Phe)	XL	Hemi	1	PM2, PP3, PM5
*EDA*	c.1133 C > G (p.Thr378Arg)	XL	Hemi	1	PM2, PP3, PM5
*EDA*	c.1142G > A (p.Gly381Glu)	XL	Hemi	1	PM2, PP3, PM5
*EDA*	c.1144G > A (p.Ala382Thr)	XL	Hemi	1	PM2, PP3
*EDARADD*	c.61 + 1156G > T (c.31 + 1G > T for NM_080738.4)	AR?	Homo	1	PM2, PVS1
*EDAR*	c.1208 C > T (p.Thr403Met)	AR?	Homo	1	PM2, PP3
*EDAR*	c.1295T > C (p.Leu432Pro)	AD?	Hetero	1	PM2, PP3
*EDAR*	c.405 C > G (p.Cys135Trp)	AR?	Homo	1	PM2, PP3
*EDAR*	c.265 C > T (p.Arg89Cys)	AD for NSTA?	Hetero	1 (both mutations in one patient)	PM2, PP3
*WNT10A*	c.1162 C > T (p.Arg388Cys)				PM2, PP3
*WNT10A*	c.644G > T (p.Ser215Ile)	AR	Homo	1	PM2, PP3
*WNT10A*	c.274G > A (p.Ala92Thr)	AR	Homo	1	PM2, PP3

An analysis of sex-specific findings revealed that the cause of HED could be identified in 61.5% of the female probands (i.e., 40 out of 65 cases). The majority of the detected variants in affected females were heterozygous changes in *EDA* (23 out of 40, 57.5%) and recessive variants in *WNT10A* (11 out of 40, 27.5%); alterations in *EDAR* were also detected (6 out of 40, 15.0%). In males with HED (196 subjects), a causative mutation was identified in 143 cases (72.9%). The vast majority of these changes were in the *EDA* gene (*n* = 132, 92.3%), followed by *EDAR* (*n* = 6, 4.2%) and *WNT10A* (*n* = 5, 3.5%).

### *EDA* gene

A total of 155 causative variants were identified in the *EDA* gene, representing 84.7% of all detected pathogenic and likely pathogenic variants. Of these, 136 were sequence variants detected by Sanger sequencing, and 19 were gross rearrangements identified by MLPA analysis (Fig. 2). While 50 variants have been previously reported (Supplementary Table 1) in literature, 41 are novel and have never been reported before (Table 2).

**Table 2:** Undescribed *EDA* variants, classified as pathogenic or likely pathogenic according to ACMG criteria

*EDA* exon	*EDA* Variant	State	Times detected	ACMG criteria
1	c.1A>T (p.Met1Leu)	Hemi, Hetero	2	PS1, PS2, PM2, PP3
1	c.5dup (p.Tyr3LeufsTer97)	Hetero	1	PVS1, PM2, PP3
1	c.55G > T (p.Glu19Ter)	Hemi	1	PVS1, PM2, PP3
1	c.66_78del (p.Gln23ValfsTer30)	Hemi	1	PVS1, PM2, PP3
1	c.133del (p.Gly45ValfsTer12)	Hemi	1	PVS1, PM2, PP3
1	c.170dup (p.Leu58ValfsTer42)	Hetero	1	PVS1, PM2, PP3
1	c.175_177del (p.Cys59del)	Hemi	1	PM2, PM4, PP3, PP1
1	c.294_296delTGAinsCATCACCAGTCACCTT (p.Asp99IlefsTer18)	Hemi	1	PVS1, PM2, PP3
1	c.358del (p.Glu120LysfsTer17)	Hemi	1	PVS1, PM2, PP3
	dup ex 1 (hg38) g.(68752763_68752820)_(68752994_68753051)dup	Hemi	1	PVS1, PM2, PP3
2	c.407T > A (p.Leu136Ter)	Hemi	1	PVS1, PM2, PP3
2	c.412_413del (p.Phe138LeufsTer4)	Hemi	1	PVS1, PM2, PP3
	del ex2-8 (hg38) g.(69093262_69093334)_(69174991_69175060)del	Hemi	1	PVS1, PM2, PP3
	del ex3-5 (hg38) g.(69159669_69159741)_(69166060_69166132)del	Hemi	1	PVS1, PM2, PP3
4	c.[538G > T;546_547delinsCT; ] (p.[Gly180Ter; Gly183Ter)	Hemi	1	PVS1, PM2, PP3
4	c.546_582del (p.Gly183AspfsTer85)	Hemi	1	PVS1, PM2, PP3
4	c.549_671del123 (p.Asn185_Pro225del)	Hemi	1	PM2, PP3 (smaller deletions are described as pathogenic)
4	c.563_676del114 (p.Pro188_Pro225del)	Hemi	1	PM2, PP3 (smaller deletions are described as pathogenic)
4	c.569_585del17 (p.Pro193ThrfsTer41)	Hemi	1	PVS1, PM2, PP3
4	c.576_592del (p.Pro193ThrfsTer41)	Hemi	1	PVS1, PM2, PP3
4	c.589del (p.Gln197ArgfsTer83)	Hemi	1	PVS1, PM2, PP3
4	c.662_696del (p.Pro222Thrfs*6)	Hemi	1	PVS1, PM2, PP3
4	c.671G > A (p.Gly224Asp)	Hemi	1	PM2, PM5, PP3, PP1
4	c.686del (p.Pro229LeufsTer51)	Hetero	1	PVS1, PM2, PP3
	del ex4 (hg38) g.69164541_69164646del	Hemi	1	PVS1, PM2, PP3
5	c.728del (p.Thr243IlefsTer37)	Hemi	1	PVS1, PM2, PP3
6	c.770G > T (p.Gly257Val)	Hemi	1	PS2, PM2, PP3
7	c.820T > C (p.Trp274Arg)	Hemi	1	PS1, PM2
7	c.839_847delinsTT (p.Asn280Ilefs*26)	Hemi	1	PVS1, PM2, PP3
7	c.880G > A (p.Glu294Lys)	Hemi	1	PS2, PM2, PP3
7	c.892_924 + 3del (p.?, splicing)	Homo/Hemi	1	PVS1, PM2, PP3
8	del ex8 (hg38) g.(69171962_69172022)_(69174991_69175060)del, hg38	Hemi	1	PVS1, PM2, PP3
8	c.928T > C (p.Tyr310His)	Hemi	1	PS2, PM2, PP3
8	c.962delA (p.Glu321GlyfsTer53)	Hemi	1	PVS1, PM2, PP3
8	c.983 C > A (p.Pro328His)	Hemi	1	PM2, PP3, PS2
8	c.1041_1042del (p.Thr348ArgfsTer20)	Hemi	1	PVS1, PM2, PP3
8	c.1043 C > A (p.Thr348Asn)	Hemi	1	PS2, PM2, PP3, PM5
8	c.1075 A > C (p.Lys359Gln)	Hemi	2	PS4, PM2, PP3
8	c.1112T > C (p.Ile371Thr)	Hetero	1	PS2, PM2, PM5, PP3
8	c.1115_1117del (p.Asn372del)	Hemi	1	PM2, PP3, PM4, PP1
8	c.1117A>G (p.Met373Val)	Hemi	2	PS4, PM2, PM5, PP3

The *EDA* gene spectrum revealed several recurrent mutations. The most notable were alterations affecting residues Arg153, Arg155, and Arg156, which collectively accounted for 23.8% (37/155) of all pathogenic variants. Other recurrent findings included deletions of more than 10 base pairs in exon 4, representing 10.3% (16/155) of variants, and gross deletions/duplications, which constituted 12.2% of all *EDA* pathogenic alterations (19/155).

Strikingly, four patients had complex genotypes. One boy had a c.[538G > T;546_547delinsCT] (p.[Gly180Ter; Gly183Ter]) alteration, which marks a rare co-occurrence of two nonsense mutations in close proximity within the same gene. Another boy harbored a c.-3_57del60 (p.Met1_Glu19del) mutation in the *EDA* gene, concurrently with a duplication in exon 1 of the *WNT10A* gene, which likely aggravated his dental phenotype. His clinical features included thin, xerotic skin, hyperpigmented periorbital regions, and scalp hypotrichosis. The dental findings were particularly notable, with conical-shaped maxillary teeth and a markedly reduced permanent dentition: only five permanent tooth buds were identified (four maxillary and one mandibular). The nails appeared normal.

Another girl was heterozygous for two *EDA* gene mutations, [1A>T(;)242C>T] (p.[Met1Leu(;)Ser81Leu]), which were initially classified as variants of uncertain significance (VUS). Their pathogenicity required subsequent reassessment, as biallelic *EDA* variants are exceptionally rare in females. Further c.1A>T (p.Met1Leu) was revealed to be *de novo* and thus likely pathogenic, whereas c.242C>T (p.Ser81Leu) was detected to be inherited from her unaffected mother and referred to her asymptomatic brother as well; thus, it was classified as likely benign.

We also report a family exhibiting intrafamilial phenotypic variability of HED among affected females, correlated with their genotypes. One girl, who was hemi-/homozygous for the c.892_924 + 3del mutation in *EDA*, presented with a severe clinical HED phenotype, whereas her heterozygous sister displayed only mild manifestations of HED.

Furthermore, in one family, a female with features of HED was found to carry the c.206G > T (p.Arg69Leu) heterozygous variant in the *EDA* gene, which is annotated in the HGMD database (CM960503) as disease-causing. However, according to the internal RCMG database, the same variant was also identified in 39 male patients examined by a clinical geneticist at RCMG, all of whom lacked any clinical manifestations of HED and had other primary indications for WES/WGS. Consequently, c.206G > T (p.Arg69Leu) in *EDA* should be reclassified as likely benign. The causative HED variant in this female patient, however, remains unidentified, and she has therefore been referred for WGS.

In 73 families, we were able to analyze both the probands and their parents. The pathogenic *EDA* variant was *de novo* in 28.8% (21/73) of cases and inherited in 71.2% (52/73). In the inherited cases, the variant was transmitted from mother in male probands or from either parent in female probands.

Almost all males with *EDA* mutations who were examined by a clinical geneticist at the RCMG were diagnosed with the classic XLHED phenotype with variable expressivity. This included varying degrees of sparse hair, reduced sweating ability, nail dystrophy and dental anomalies. However, several male patients with c.947A>G (p.Asp316Gly), c.1013C>T (p.Thr338Met), and c.1069C>T (p.Arg357Trp) mutations did not experience hypohidrosis and hypotrichosis but predominantly exhibited multiple-tooth agenesis.

Female probands with *EDA* mutations consistently presented milder signs of HED. Notably, heterozygous variants in *EDA* can result in mild clinical manifestations of HED in women, even in the absence of skewed X chromosome inactivation patterns [14]. A comparison of blood X-chromosome inactivation (XCI) profiles between 23 females with HED manifestations and 24 asymptomatic females, all of whom carried pathogenic *EDA* variants, showed no statistically significant difference (p-value = 0.13). In family ED53, despite both presenting with HED features, two females showed contrasting XCI profiles: one with skewing toward the mutant *EDA* allele and the other toward the wild-type allele. Three females (ED148, ED222.1, and ED276) who exhibited HED clinical signs and skewed X-chromosome inactivation patterns were excluded from the analysis because of the inability to determine which allele underwent inactivation (Supplementary Table 2).

### *EDAR* gene

Disease-causing mutations in the *EDAR* gene were responsible for HED in 12 unrelated patients, accounting for 6.5% of all molecularly confirmed cases (Table 3). Among the causative mutations in the *EDAR* gene, the most prevalent were (1) the homozygous variant c.71 C > A (p.Ala24Asp) (3 patients, 6 out of 24 chromosomes, 25%) and (2) the recessive variant c.1135G > A (p.Glu379Lys) (3 patients, 4 out of 24 chromosomes, 16.6%) and (3) the heterozygous variant c.1072 C > T (p.Arg358Ter) (3 out of 12 patients, 25%). Given this distribution, these pathogenic variants should be considered recurrent in *EDAR* among patients from the Russian Federation.

**Table 3:** All detected pathogenic and likely pathogenic *EDAR* variants

ID, sex	MOI	Variant 1 (*R*- reported/ *N*- novel)	Variant 2 (*R*- reported/ *N*- novel)	Times detected
ED205 (M) ED209 (M) ED280 (M)	AR	c.71 C > A (p.Ala24Asp), R	c.71 C > A (p.Ala24Asp), R	3
ED44 (M)	AR	c.1135G > A (p.Glu379Lys), R	c.126del (p.Leu43CysfsTer60), R	1
ED237 (M)	AR	c.1135G > A (p.Glu379Lys), R	c.1135G > A (p.Glu379Lys), R	1
ED77 (F)	AR	c.1135G > A (p.Glu379Lys), R	c.266G > A (p.Arg89His), R	1
ED138 (M), ED177 (F), ED241 (F)	AD	c.1072 C > T (p.Arg358Ter), R	-	3
EDA271 (F)	AD	c.1098dup (p.Ala367ArgfsTer2), N	-	1
ED354 (M)	AD	c.1144_1147del (p.Gly382Ter), N	-	1
ED169 (F)	AR	Entire *EDAR* deletion, N	c.1123C>T (p.Arg375Cys), N	1

### *EDARADD* gene

No apparent pathogenic mutations were detected in the *EDARADD* gene in patients of either sex. However, in one case, a female with classic HED-phenotype was found to have a homozygous/hemizygous variant c.61 + 1156G > T in *EDARADD* (NM_145861.3; chr1:236395661G > T, hg38). Although this variant should initially be classified as a VUS, it is considered to affect the canonical splice site (c.31 + 1G > T) when mapped to another *EDARADD* transcript - NM_080738.4 (ENST00000359362.6). This transcript encodes the functional EDARADD isoform B (205 aa), which, along with isoform A (215 aa) activates the NF-κB pathway [15]. Therefore, we believe this variant is likely to be reclassified as disease-causing after functional mRNA analysis.

**Fig. 3:**
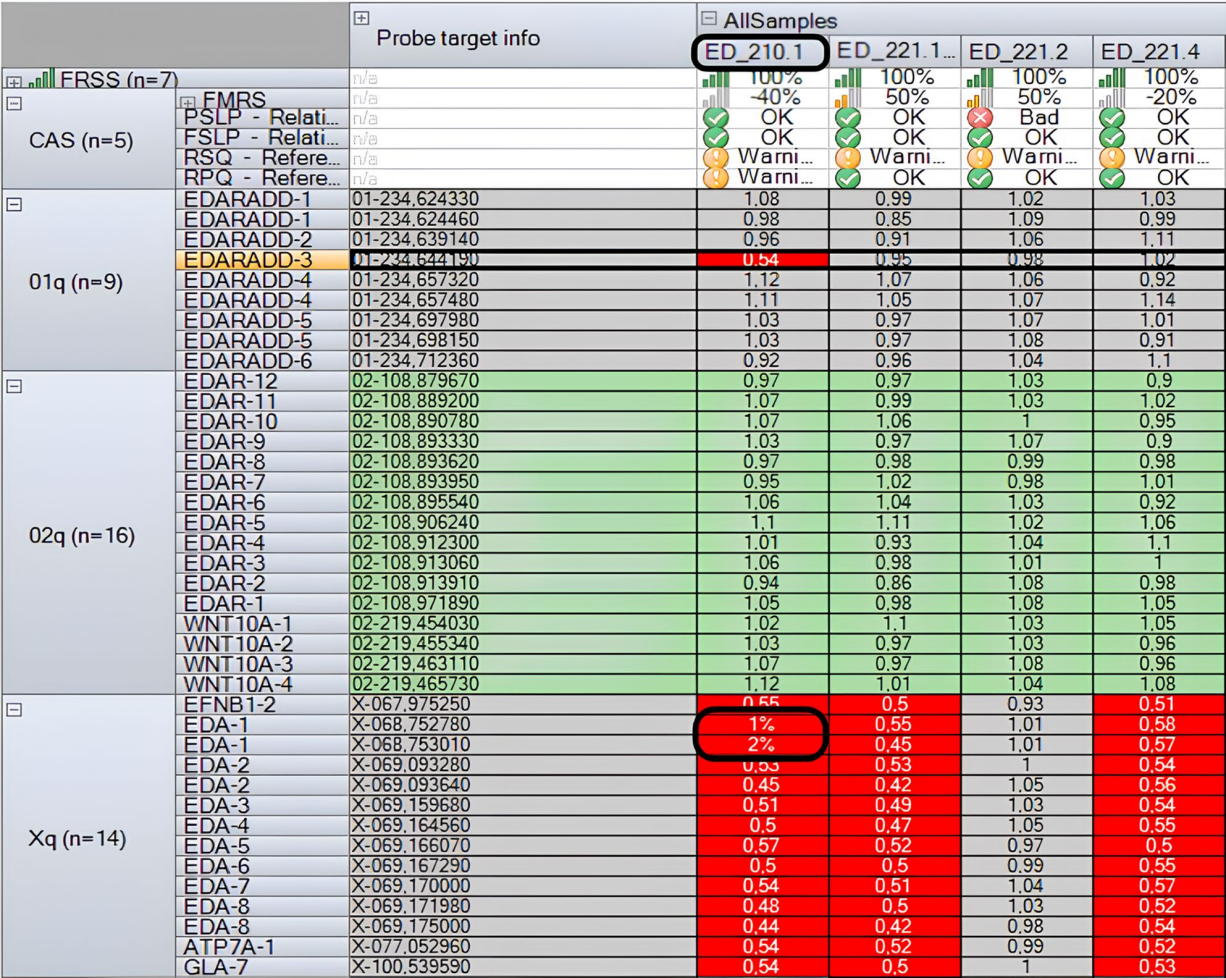
Plot of the MLPA analysis (Coffalyser), demonstrating the reduced signal intensity in exon 3 of the *EDARADD* gene, as well as in exon 1 of the *EDA* gene in sample ED_210.1

**Fig. 4:**
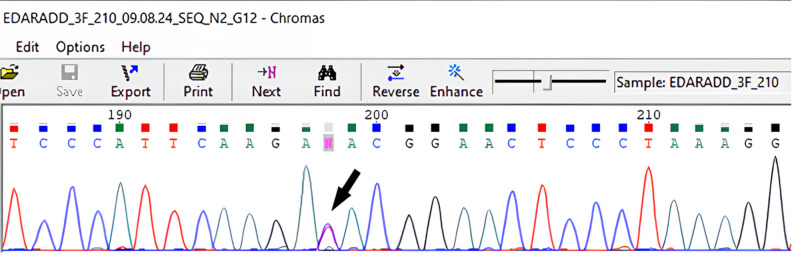
The sequencing chromatogram shows a benign substitution c.144T > C (p. Asp48=), which interferes with the hybridization of the *EDARADD* exon 3 MLPA probe (SALSA^®^ MLPA^®^ Probemix P183 EDA-EDAR-EDARADD)

In another case, during MLPA analysis, a reduced signal intensity was observed for exon 3 of the *EDARADD* gene (Fig. 3). Subsequent Sanger sequencing revealed that this resulted from a benign polymorphism, c.144T > C (p.Asp48=), on one allele, which interfered with probe binding (Fig. 4). Therefore, this finding was not interpreted as a true deletion. Moreover, a deletion of *EDA* exon 1 was identified in this patient (ED_210.1) (Fig. 3).

### *WNT10A* gene

*WNT10A* is associated with non-syndromic tooth agenesis (NSTA) or oligodontia of varying severity in individuals with either heterozygous or biallelic pathogenic alterations. However, in cases of HED or OODD, both alleles must be affected. In patients with OODD, the primary complaints typically involve misshapen teeth and a reduced number of teeth, as well as dystrophic nails, whereas other manifestations—such as dry skin, hypohidrosis, and poor hair growth—are considered secondary concerns.

The *WNT10A* gene is the second most frequently implicated HED gene in Russia (8.8% of cases, *n* = 16). It is also characterized by the presence of common pathogenic variants in compound-heterozygous or homozygous states (Table 4), including c.321 C > A (p.Cys107Ter) (19 out of 32 chromosomes, 59,4%), c.682T > A (p.Phe228Ile) (4 out of 32 chromosomes, 12,5%), and c.337 C > T (p.Arg113Cys) (2 out of 32 chromosomes, 6,3%). Although the c.682T > A (p.Phe228Ile) variant is found in a homozygous state in 22 individuals in the gnomAD v3.1.2 database with an allele frequency of 1.406%, its heterozygous frequency is 10 times higher in individuals with selective tooth agenesis [16] and is significantly enriched in individuals with ED manifestations in our cohort. Therefore, it should be considered a hypomorphic variant, that can lead to manifestations of ED when combined with a pathogenic or likely pathogenic variant on the other allele. We also identified two novel variants in *WNT10A*: a frameshift mutation, c.1087_1100del (p.Asn363ArgfsTer60), and a duplication of exon 1.

**Table 4:** All detected *WNT10A* pathogenic or likely pathogenic variants

ID, sex	Variant 1 (*R*- reported/ *N*- novel)	Variant 2 (*R*- reported/ *N*- novel)	Times detected
ED188 (M), ED335 (F), ED249 (F), ED307 (F), ED85 (F), ED 41,679/2024 (F)	c.321C>A (p.Cys107*), R	c.321C>A (p.Cys107*), R	6
ED173 (F), ED270 (F), ED272 (M)	c.321C>A (p.Cys107*), R	c.682T>A (p.(Phe228Ile), R	3
ED153 (F), ED214 (M)	c.321C>A (p.Cys107*), R	c.337C>T (p.Arg113Cys), R	2
ED140 (M)	c.321C>A (p.Cys107*), R	c.391G>A (p.Ala131Thr), R	1
ED375 (F)	c.321C>A (p.Cys107*), R	c.742C>T (p.Arg248*), R	1
ED277 (F)	c.1087_1100del (p.Asn363ArgfsTer60), N	c.208C>T (p.Arg70Trp), R	1
ED126 (F)	c.826T>C (p.Cys276Arg), R	c.682T>A (p.(Phe228Ile), R	1
ED353 (M)	dup 1 ex (hg38) g.219454020_219454085dup, N	c.-3_57del60 (p.Met1_Glu19del) in *EDA*	1

## Discussion

The *EDA*,* EDAR*,* EDARADD*, and *WNT10A* genes are the four primary genes responsible for the development of HED. To date, unfortunately, few large cohort studies have been conducted on HED patients, making comprehensive comparative analysis particularly challenging.

An analysis of the distribution of pathogenic variants in these genes across countries with available data revealed a clear pattern: *EDA* gene mutations are predominant in all regions, including Russia. However, in Turkey, *EDA* and *EDAR* mutations are detected at equal frequencies [17]. This phenomenon has been attributed to the high incidence of consanguineous marriages but may also be influenced by the small cohort size (17 families) [17]. In most countries with available data, such as Mexico [18], Egypt [19], Turkey [17], Germany [20], France [21], and Korea [22], the second most affected gene is either *EDAR* or *EDARADD*. In contrast, among Russian patients, *WNT10A* ranks as the second most frequently mutated gene, like as in Spain [23]. Notably, in the Russian population, no *EDARADD* pathogenic mutations were identified among 261 unrelated HED patients, whereas in other countries, variants in this gene are relatively common.

**Fig. 5:**
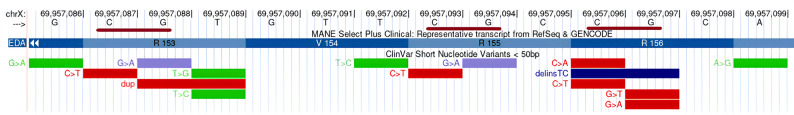
The clustering of mutations at *EDA* furin cleavage site residues (Arg153, Arg155, Arg156) is driven by 5-methylcytosine deamination within this CpG-enriched region, - a well-established hotspot for C-to-T transitions

Among the common mutations in the *EDA* gene across all countries with published HED cohorts, the predominant changes involve the amino acids Arg153, Arg155, and Arg156, similar to those observed in Russia [17–24]. This phenomenon can likely be explained by a CpG-rich sequence in exon 2, specifically at arginine codons 153, 155, and 156 (CGT, CGC, and CGC) (Fig. 5). When this region undergoes methylation, it becomes prone to 5-methylcytosine deamination and C-to-T transition [25]. Large deletions in exon 4 of the *EDA* gene are also commonly observed in many countries, including Russia.

However, the spectrum of frequent mutations in the *EDAR* gene varies slightly. For example, the c.1135G > A (p.Glu379Lys) variant is absent among the recurrent mutations reported in European, Asian and Arab countries [26]. In Turkey, as in Russia, the c.71 C > A (p.Ala24Asp) variant is among the most frequently observed *EDAR* mutations [17], whereas in Europe, the c.1072 C > T (p.Arg358Ter) variant is the most common [27].

Several previous studies have demonstrated the presence of recurrent mutations in the *WNT10A* gene [28–30]. In a cohort of European patients with HED, the nonsense variant p.Cys107* was found in 50% of cases (12 out of 24 alleles), while the missense variant p.Phe228Ile was present in 25% (6 out of 24 alleles) [28]. This distribution is consistent with the data obtained in the present work. A similar trend was observed in a Polish study, where both variants were most frequently identified in a heterozygous state in patients with NSTA [29]. Furthermore, research established that these variants, together with c.391G > A (p.Ala131Thr), accounted for 57.1% of all pathogenic alleles in Italian patients with *WNT10A*-associated ED [30]. These findings further confirm their significant contribution to the genetic architecture of the disorder across European populations. Notably, no *WNT10A* mutations were identified in the Egyptian cohort [19], while in Turkey, the c.433G > A (p.Val145Met) variant was predominant, rather than the aforementioned variants [17].

We identified 46 novel likely pathogenic variants, including 41 in *EDA*, 4 in *EDAR* and 2 in *WNT10A*. Among these variants, 37 mutations were plain loss-of-function (LOF) variants with low (or no) population allele frequencies. However, 9 *EDA* variants were missense mutations and initially posed significant challenges in interpretation; thus, their pathogenicity was ultimately determined via broad segregation analysis.

Unfortunately, many of the mutations revealed in HED genes remain classified as variants of uncertain clinical significance (VUS), posing considerable challenges to diagnosis and interpretation. In our cohort, 30 mutations were identified as VUS; although we believe that they are likely causative, further tests are needed for reassessment of their disease impact.

This issue is particularly evident in the *EDA* gene, where a large portion of variants in male patients are inherited from their mothers (at least 71.2%), who seem mostly unaffected. Further analysis of the maternal X chromosome inactivation pattern to detect potential skewed lyonization is generally not informative. First, conventional analysis of maternal DNA alone can identify an imbalance in allele expression but cannot be used to determine whether the expressed allele is wild type or mutant. Second, our data indicate that XCI patterns in blood do not correlate with the severity of HED clinical manifestations, probably due to the extremely low expression level of *EDA* in blood (0.16 TPM, GTEx database) (GTEx). While analyzing the pattern of X chromosome inactivation in female *EDA*-carriers and female patients with *EDA*-associated ectodermal dysplasia, we detected no strong correlation between the presence of clinical manifestations and skewed X chromosome inactivation patterns. Spanish colleagues reached a similar conclusion, showing that a significant portion of female probands with an *EDA*-causative variant exhibit a random pattern of X inactivation [23]. Another study also confirmed no evidence of preferential X inactivation in female XLHED patients and reported no distinct correlation between XLHED-related phenotypic features and X inactivation patterns [14]. Furthermore, they reported that skewed X inactivation favoring the mutated allele was not associated with more severe phenotypes.

The determination of the pathogenicity of VUS can be achieved either through segregation analysis involving a larger number of affected and healthy relatives or through functional analysis. However, in Russia, where families typically have only one or two children [31], large-scale cosegregation analysis is feasible in only a limited number of cases.

**Fig. 6:**
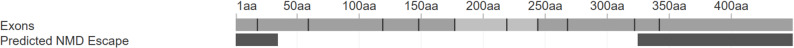
Predicted NMD escape in the *EDAR* gene according to the DECIPHER database

Challenges also arise in determining the pathogenicity of novel mutations in the *EDAR* gene, as it follows both autosomal recessive and autosomal dominant inheritance patterns. Consequently, the identification of a new variant on a single allele does not necessarily rule out heterozygous carrier status, particularly since the correlation between mutation location, type, and inheritance mode is still being established. Variants that result in a premature stop codon in mRNA are typically considered to have a recessive effect, as the mutant mRNA is likely degraded via nonsense-mediated mRNA decay (NMD). However, mutations that produce premature stop codons in the last exon, such as p.Glu354X and p.Arg358X, do not activate NMD. Instead, the loss of the death domain (DD) can lead to a dominant-negative effect, thereby causing the autosomal dominant (AD) form of *EDAR*-associated HED [27] (Fig. 6). Almost all missense mutations located in the functional ligand binding domain (LBD) (Fig. 1) are considered recessive. However, one report described a mildly affected female with heterozygous *EDAR* mutation p.Arg89His. Based on this case, the authors hypothesized that all missense mutations might exhibit a dominant effect with significant phenotypic variability [27].

Regarding the domain structure of *EDA*, it is important to note that some mutations do not lead to the classic manifestations of HED but instead result in NSTA. Several studies have established that mild missense mutations in *EDA*, which cause only oligodontia, are predominantly (80%) located in the TNF domain of the ectodysplasin-A protein [32, 33] (Fig. 1). This phenotypic variation has been attributed to the residual EDA receptor-binding capacity of certain NSTA-causing mutations, in contrast to the complete loss of binding ability observed in HED-causing *EDA* mutations. Our findings support this observation, as male patients with c.947 A > G (p.Asp316Gly), c.1013 C > T (p.Thr338Met), and c.1069 C > T (p.Arg357Trp) mutations did not experience hypohidrosis or profound hypotrichosis but exhibited multiple-tooth agenesis.

In the Russian population, genetic testing for suspected HED should first target the *EDA* gene in both sexes, followed by *WNT10A* point mutation analysis in female patients.

Understanding the distribution of genes associated with HED, particularly the frequency of *de novo* variants in the *EDA* gene (28,8%), also promotes the reconsideration of indirect prenatal testing. This remains important even in cases where rapid identification of the causative variant is not possible.

## Conclusion

In this study, we present the largest HED cohort to date, reporting on HED-cases in both the Russian Federation and globally. Our findings significantly expand the mutational spectrum of HED-causing genes, particularly with the identification of 46 novel mutations.

We do not recommend analyzing X inactivation patterns in blood, as it does not clarify the pathogenicity of *EDA* variants of uncertain significance and does not predict HED severity in women. Our study highlights the genetic heterogeneity of HED. For cases without a molecular diagnosis, WGS could provide valuable insights and help identify key proteins involved in HED pathogenesis, offering directions for future research.

### Web resources

NGS-Data: https://ngs-data-ccu.epigenetic.ru/ .

RUEXAC: https://ngs-data-ccu.epigenetic.ru/vcfdb/ .

gnomAD: https://gnomad.broadinstitute.org/ .

Online Mendelian Inheritance in Man (OMIM): https://www.omim.org/ .

GTEX: https://gtexportal.org/home/gene/EDA .

Decipher: https://www.deciphergenomics.org/.

REVEL: https://genome.ucsc.edu/cgi-bin/hgTrackUi?db=hg19%26g=revel.

PolyPhen-2: http://genetics.bwh.harvard.edu/pph2/ .

SIFT Indel: https://sift.bii.a-star.edu.sg/www/SIFT_indels2.html .

PROVEAN: https://provean.jcvi.org/ .

M-CAP: http://bejerano.stanford.edu/mcap/.

CADD: https://cadd.gs.washington.edu/ .

Mutation Assessor: https://mutationassessor.org/ .

Mutation Taster: http://www.mutationtaster.org/ .

SpliceAI: https://spliceailookup.broadinstitute.org/ .

Human Splicing Finder http://www.umd.be/HSF/.

## Supplementary Information

Below is the link to the electronic supplementary material.

Supplementary Material 1

Supplementary Material 2

## Data Availability

The datasets used and/or analyzed during the current study are available from the corresponding author upon reasonable request.
